# Impact of body size, nutrition and socioeconomic position in early life on the epigenome: a systematic review protocol

**DOI:** 10.1186/s13643-017-0521-8

**Published:** 2017-07-05

**Authors:** Jane Maddock, Wahyu Wulaningsih, Rebecca Hardy

**Affiliations:** MRC Lifelong Health and Ageing at UCL, 33 Bedford Place, London, WC1B5JU UK

**Keywords:** Epigenome, Epigenetics, Early life, Body size, Growth, Nutrition, Socioeconomic position

## Abstract

**Background:**

Body size, nutrition and socioeconomic position (SEP) in early life have been associated with a range of later life health outcomes. Epigenetic regulation is one mechanism through which these early life factors may impact later life health. The aim of this review protocol is to outline procedures to document the influence of body size, nutrition and SEP in early life on the epigenome.

**Methods:**

MEDLINE, Embase and BIOSIS will be systematically searched using pre-defined keywords. Additional studies will be identified through manual searching of reference lists. Two independent researchers will assess the eligibility and quality of each study, with disagreements being resolved through discussion or a third reviewer. Studies will be included if they have epigenetic markers measured either at the same time as, or after, the early life exposure and, have a measure of body size, nutrition or SEP in early life (up to 12 years), are in the English language and are from a sample of community-dwelling participants.

**Discussion:**

This protocol will be used to collate the evidence for the effect of early life factors on the epigenome. Findings will form a component of a wider research study examining epigenetic responses to exposures in early life and over the life course and its impact on healthy ageing using data from population-based cohort studies.

**Systematic review registration:**

PROSPERO CRD42016050193

**Electronic supplementary material:**

The online version of this article (doi:10.1186/s13643-017-0521-8) contains supplementary material, which is available to authorized users.

## Background

The extensive growth and development that occurs in utero and childhood marks a sensitive period during which external factors can elicit biological changes which programme an individual’s ability to maintain health or increase risk of later life disease [1–3]. Body size, nutrition and socioeconomic position (SEP) at birth and during childhood are commonly studied characteristics which have been strongly linked with later life health [4–9].

There are a variety of mechanisms through which these early life factors may impact later life health [2, 4, 7, 10]. Epigenetic regulation is one such mechanism [2, 11]. Epigenetics refers to alterations in gene expression. Epigenetic modifications do not alter the sequence of DNA, but can regulate how that DNA is expressed and ultimately, how the gene functions. Epigenetic modifications include histone modifications and DNA methylation amongst others [12]. These malleable epigenetic signals are essential for development and cell proliferation but can also occur in response to environmental stimuli [13]. Evidence from animal studies and a limited number of human studies suggests that early life exposures can modify several epigenetic signatures in a multitude of genes [11, 14].

Understanding which early life exposures evoke changes in the epigenome, and identifying the genomic regions in which these changes are occurring will support efforts to maintain health and prevent disease in later life. Furthermore, since epigenetic changes are potentially modifiable, a greater understanding of the specific changes induced by early life factors can elucidate potential targets for intervention strategies.

Since this is a relatively new area of research, most evidence examining the epigenetic effect of early life factors have come from animal and exploratory studies incorporating a variety of early life exposures and applying different analytical methods. Although there has been some review of the role of early life factors on the epigenome in humans [15], to the best of our knowledge, there has been no systematic review of the existing literature examining body size, nutrition and social exposures in relation to epigenetic signals. This review will collate human studies examining the impact of these early life exposures on epigenetics measured at the same time or any age subsequent to the exposure.

### Aim

The overall aim of the review is to examine the epigenetic effect of the following factors:

i. Body size and growth in early life
ii. Nutrition during pregnancy and early life
iii. Markers of socioeconomic position in early life

## Methods/design protocol registration

This study protocol is registered with the PROSPERO database (registration number: CRD42016050193) and has been reported using the Preferred Reporting Items for Systematic Reviews and Meta-Analyses Protocols (PRISMA-P) checklist, included as Additional file 1.

### Key terms

For the purposes of this review, early life is defined as the period including prenatal, infancy, early and middle childhood, up to 12 years to capture the pre-adolescence period. Full definitions of each of each key variable used in the review can be found in Table 1.

**Table 1:** Definitions of key variables included in review

Term	Description
Epigenetics	Any recognised indicator of DNA methylation or histone modification, measured in any tissue
Body size	Any measure of body size including: weight, height, BMI, head circumference and growth (or change) in any of these measurements at birth and in early life
Nutrition	Any measure of maternal nutrition, supplement use and/or diet during pregnancy, breastfeeding/formula, weaning practices and nutrition/diet of the child in early life measured using dietary questionnaires and/or objectively measured nutritional biomarkers
Socioeconomic position (SEP)	Any recognised indicator of SEP within society, including occupation, education, income, occupational or social class, poverty and household overcrowding, as defined in reference [16], measured during early life

### Eligibility criteria

Papers will be included if they meet the following criteria:

- Original studies published in peer-reviewed journals
- Epigenetic markers measured either at the same time or any age subsequent to the early life exposure (i.e. epigenetics can be measures in infancy, childhood, adolescence or adulthood)
- Current, prospective report or retrospective recall of indicators of body size (see Table 1)
- Current, prospective report or retrospective recall of nutrition and/or diet (see Table 1)
- Current, prospective report or retrospective recall SEP (see Table 1)
- English language
- Non-clinical samples

We will not include:

- Studies not meeting inclusion criteria
- Reviews
- Studies based on animals
- Studies only assessing the effect of adulthood exposures on epigenetic markers
- Studies assessing the effect of epigenetic markers before early life exposures
- Samples with a specific clinical condition

### Search strategy

Using OvidSP as the database interface, a joint electronic search on MEDLINE and Embase will be conducted. The BIOSIS database will be searched using ISI Web of Science. The search will use free-text search terms (Table 2) with truncations to allow for different spellings, proximity operators (‘adj’ in OvidSP, ‘NEAR’ in ISI Web of Science) and joined using Boolean logic (“AND”, “OR”). The reference lists’ of relevant reviews, all included papers and their ISI citation index (via Web of Science) will be searched for studies meeting inclusion criteria. Eligible studies identified will be combined with the electronic search results.

**Table 2:** Search terms

Epigenetics	
1	(epigen* )
2	(EWAS)
3	(Methylation)
4	(DNA adj5 methyl*)
5	(differential adj1 methylation)
6	(global adj1 methylation)
7	(DNA adj5 hypermethyl*)
8	(DNA adj5 hypomethyl*)
9	(gene* adj5 methyl*)
10	Gene Expression Regulation
11	(methyl* adj5 region)
12	(histone adj1 modif*)
13	1 OR 2 OR 3 OR 4 OR 5 OR 6 OR 7 OR 8 OR 9 OR 10 OR 11 OR 12
Body size/ growth	
14	((premature or pre-mature or preterm or pre-term) adj2 (birth or infant* or child* or neonat*))
15	(gestation* adj2 age)
16	(weight or body-size or bmi or height or length or head-circumference)
17	((grow*) adj3 (fet* or neonat* or prenatal* or pre-natal* or intrauterine or in-utero or postnatal or post-natal or birth* or infant* or child* or early-life or earlylife))
18	(fet* or neonat* or prenatal* or pre-natal* or intrauterine or in-utero or postnatal or post-natal or birth* or infant* or child* or early-life)
19	16 OR 17
20	18 AND 19
21	14 OR 15 OR 20
Nutrition	
22	(nutrition adj3 (maternal or mother* or pregn* or fet* or neonat* or prenatal* or pre-natal* or intrauterine or in-utero))
23	((nutrition or diet or wean*) adj3 (postnatal or post-natal or infant* or child* or early-life))
24	(breastfe* or breast-fe*)
25	(formula adj3 (infant* or child* or early-life))
26	22 OR 23 OR 24 OR 25
Socioeconomic position	
27	((occupation* or education*) adj3 (father* or mother* or parent*))
28	((income or manual) adj3 (father* or mother* or parent*))
29	((social class or social status) adj3 (father* or mother* or parent* or child* or early-life))
30	((socioeconomic or socio-economic) adj3 (father* or mother* or parent* or child* or early-life))
31	((deprivation or poverty) adj3 (child* or early-life))
32	((overcrowding) adj3 (child* or early-life))
33	27 OR 28 OR 29 OR 30 OR 31 OR 32
Combining results	
34	21 OR 26 OR 33
35	13 AND 33

### Study selection

Results of the searches will be merged and stored in the reference manager, EndNote. All duplicate citations will be removed. All abstracts will be screened for eligibility by two independent researchers (from JM, WW and RH). The full text of all potentially eligible papers will then also be double screened and reasons for their exclusion will be documented. Disagreements about the paper’s eligibility will be resolved through discussion and if necessary, a third reviewer.

### Data extraction

Each reviewer will extract the relevant information using the data extraction form (Additional file 2).The following information will be extracted from selected papers: citation details, study details including design, country/region and sample size; participant details including age and sex; and exposure and outcome details, including details on methods used. A free-text box for recording main findings will be used because of the expected heterogeneous methods that will have been used.

### Quality assessment

Various tools for assessing the quality of observational studies such as the Downs and Black checklist [16] and Newcastle-Ottawa Scale [17] are available. However, these are not suitable for assessing the quality of epigenetic studies. The following aspects of the paper which may relate to the quality of each study will be extracted and discussed; study design, methods used to measure epigenetics, statistical analysis including adjustment of relevant confounders, recall bias, i.e. prospective or retrospective measures of early life factors and generalisability [18].

### Synthesis

Descriptive tables will be used to outline the included studies in the systematic review. Results will be grouped under the following thematic groups: (1) body size in early life; (2) nutrition in early life; 3) SEP in early life. If there is more than one exposure assessed in an individual study, they will be discussed separately where practical. If feasible, the effects of similar epigenetic analyses methods, e.g. epigenome-wide association studies/locus-specific studies and the age at which epigenetic markers were identified will also be grouped within each theme.

Due to the expected diversity in eligible studies, in terms of methods used, a meta-analysis will not be conducted [18]. Therefore, a narrative synthesis will be undertaken. The structure of the narrative synthesis will be based on guidance from Economic and Social Research Council Methods Programme [19]. This will involve preliminary synthesis, exploring relationships between and within studies, with particular focus on sources of heterogeneity in study design and characteristics between studies, and assessing the robustness of the synthesis, i.e. assessing the strength of the evidence given the methodological quality and generalisability of included papers [18, 19].

### Reporting

The findings of this systematic review will be reported in accordance with the Preferred Reporting Items for Systematic Reviews and Meta-Analyses Protocols (PRISMA-P) guidelines [20].

## Discussion

This study will systematically review the literature examining the effect of body size and growth, nutrition and socioeconomic position in early life on the epigenome. The relationship between different early life exposures and epigenetics as well as other sources of between-study heterogeneity will be explored. The strengths and limitations, and therefore potential for bias, of individual studies will be considered. Since this is a relatively new field, we anticipate that there will be a small number of studies and considerable between-study heterogeneity in terms of study design and characteristics. In addition to collating existing evidence of the effects of early life on the epigenome, this review will form a significant component of a wider research study examining epigenetic responses to exposures in early life and over the life course and its impact on healthy ageing.

## Additional files

Additional file 1: PRISMA. PRISMA-P 2015 Checklist. Preferred Reporting Items for Systematic Reviews and Meta-Analyses Protocols (PRISMA-P) guidelines. (PDF 222 kb)

Additional file 2: Data extraction. Data extraction form. Template for extracting data from selected papers. (DOCX 25 kb)

## Abbreviations

BMI: Body mass index
PRISMA: Preferred Reporting Items for Systematic Reviews and Meta-Analyses
SEP: Socioeconomic position
