# Supplementary material for: Impact of body size, nutrition and socioeconomic position in early life on the epigenome: a systematic review protocol
Source: Syst Rev. 2017 Jul 5;6:129. doi: 10.1186/s13643-017-0521-8 (PMC5499029; doi:10.1186/s13643-017-0521-8)
Supplement: Supplementary file 2 — Data extraction. Data extraction form. Template for extracting data from selected papers. (DOCX 25 kb) [file 13643_2017_521_MOESM2_ESM.docx]

**Additional file 2: Data extraction form**

| A Reference details |  |  |  |  |  |  |  |  |
| --- | --- | --- | --- | --- | --- | --- | --- | --- |
| 1^st^ Author |  | | | | | | | |
| Title of paper |  | | | | | | | |
| Journal |  | | | | | | | |
| Volume |  | | | | | | | |
| Year of publication |  | | | | | | | |
| Publication type | Paper |  | Abstract |  | Other |  | |  |
| Assessor’s name | JM |  | WW |  | RH | |  | |
| Date |  | | | | | | | |

| Yes |  | No |  |
| --- | --- | --- | --- |

B. Study included in systematic review:

| Reason(s) for exclusion (if excluded): |  |
| --- | --- |
| Ineligible exposure |  |
| Ineligible outcome |  |
| Ineligible sample i.e. non-clinical sample |  |
| Review article |  |
| Duplicate |  |
| Exposure not in early childhood |  |
| Epigenetic marker measured before early life exposure |  |
| Other please specify: |  |

| C. Study details |  |  |  |  |  |  |  |  |  |  |  |  |  |
| --- | --- | --- | --- | --- | --- | --- | --- | --- | --- | --- | --- | --- | --- |
| Name of study/cohort |  | |  |  | |  | |  |  |  |  | |  |
| Design | Cross-sectional^1^ | |  | Prospective cohort^2^ | |  | | Other^4^ | | |  | |  |
| *If other:* |  | | | | | | | | | | | | |
| Country | US^1^ |  | | UK^2^ |  | | AUS^3^ | |  | Other^4^ | |  | |
| *If other:* |  | | | | | | | | | | | | |
| N |  | | | | | | | | | | | | |
| % Female |  | | | | | | | | | | | | |
| Other comments |  | | | | | | | | | | | | |

| D. Early life exposures used | | | |  |  |  |  |  |  |  |  |  |  |  |  |  |  |  |  |  |  |  |
| --- | --- | --- | --- | --- | --- | --- | --- | --- | --- | --- | --- | --- | --- | --- | --- | --- | --- | --- | --- | --- | --- | --- |
| Body size | | | | | | | | | | | | | | | | | | | | | | |
| Premature status | Yes |  | | No | | |  | | Measurement details: | | | | | | |  | | | | | | |
| Age at gestation | Yes |  | | No | | |  | | Measurement details: | | | | | | |  | | | | | | |
| Birthweight | Yes |  | | No | | |  | | Measurement details: | | | | | | |  | | | | | | |
| Weight in early childhood | Yes |  | | No | | |  | | Measurement details: | | | | | | |  | | | | | | |
| Height/length in early childhood | Yes |  | | No | | |  | | Measurement details: | | | | | | |  | | | | | | |
| Head circumference in early childhood | Yes |  | | No | | |  | | Measurement details: | | | | | | |  | | | | | | |
| Growth in early childhood | Yes |  | | No | | |  | | Measurement details: | | | | | | |  | | | | | | |
| Other (please specify): |  | | | | | | | | Measurement details: | | | | | | |  | | | | | | |
| How ascertained (if more than one exposure measured, list for each) | Prospectively | | | | | | |  | Retrospectively | | | | | | |  | NA | | | | |  |
| Age recorded (yrs.) (if more than one exposure measured, list for each) |  | | | | | | | | | | | | | | | | NA | | | | |  |
| Categorisation of exposure (if more than one exposure measured, list for each) | Continuous | | |  | | Categorical | | | | |  | | | Quintiles | |  | Other | |  | NA | |  |
| *If other, please specify* |  | | | | | | | | | | | | | | | | | | | | | |
| Nutrition | | | | | | | | | | | | | | | | | | | | | | |
| Maternal dietary questionnaire during pregnancy | Yes |  | No | | | |  | | Measurement details: | | | | | | |  | | | | | | |
| Maternal dietary supplements during pregnancy/lactation | Yes |  | No | | | |  | | Measurement details: | | | | | | |  | | | | | | |
| Maternal nutritional biomarker | Yes |  | No | | | |  | | Measurement details | | | | | | |  | | | | | | |
| Breastfeeding | Yes |  | No | | | |  | | Measurement details | | | | | | |  | | | | | | |
| Formula fed | Yes |  | No | | | |  | | Measurement details | | | | | | |  | | | | | | |
| Early life nutritional biomarker | Yes |  | No | | | |  | | Measurement details | | | | | | |  | | | | | | |
| Early life dietary intake (parental report) | Yes |  | No | | | |  | | Measurement details | | | | | | |  | | | | | | |
| Early life dietary supplements (parental report) | Yes |  | No | | | |  | | Measurement details | | | | | | |  | | | | | | |
| Other (please specify): |  | | | | | | | | Measurement details: | | | | | | |  | | | | | | |
| How ascertained (if more than one exposure measured, list for each) | Prospectively | | | | | | |  | Retrospectively | | | | | | |  | NA | | | | |  |
| Age recorded (yrs.) (if more than one exposure measured, list for each) |  | | | | | | | | | | | | | | | | NA | | | | |  |
| Categorisation of exposure (if more than one exposure measured, list for each) | Continuous | |  | | Categorical | | | | | | |  | | | Quintiles |  | Other |  | | NA | |  |
| *If other, please specify* |  | | | | | | | | | | | | | | | | | | | | | |
| SEP | | | | | | | | | | | | | | | | | | | | | | |
| Father’s education | Yes |  | | No | | |  | | Measurement details: | | | | | | |  | | | | | | |
| Mother’s education | Yes |  | | No | | |  | | Measurement details: | | | | | | |  | | | | | | |
| Father’s occupation | Yes |  | | No | | |  | | Measurement details: | | | | | | |  | | | | | | |
| Mother’s occupation | Yes |  | | No | | |  | | Measurement details: | | | | | | |  | | | | | | |
| Income | Yes |  | | No | | |  | | Measurement details: | | | | | | |  | | | | | | |
| Area-level deprivation index | Yes |  | | No | | |  | | Measurement details: | | | | | | |  | | | | | | |
| Other (please specify): |  | | | | | | | | Measurement details: | | | | | | |  | | | | | | |
| How ascertained (if more than one exposure measured, list for each) | Prospectively | | | | | | |  | Retrospectively | | | | | | |  | NA | | | | |  |
| Age recorded (yrs.) (if more than one exposure measured, list for each) |  | | | | | | | | | | | | | | | NA | | | | | |  |
| Categorisation of exposure (if more than one exposure measured, list for each) | Continuous | |  | | Categorical | | | | |  | | | Quintiles | | |  | Other | | |  | NA |  |
| *If other, please specify* |  | | | | | | | | | | | | | | | | | | | | | |

| E. Epigenetic outcomes |  |  |  |  |  |  |  |  |  |  |  |  |  |  |  |  |
| --- | --- | --- | --- | --- | --- | --- | --- | --- | --- | --- | --- | --- | --- | --- | --- | --- |
| Epigenetic marker | Global DNA methylation |  | Gene-specific methylation | | |  | Genome-wide methylation | | |  | Histone modification | |  | Other | |  |
| *If other please list* |  | | | | | | | | | | | | | | | |
| Methylation sites / method |  | | | | | | | | | | | | | | | |
| Tissue(s) |  | | | | | | | | | | | | | | | |
| Age(s) ascertained (yrs.) |  | | | | | | | | | | | | | | | |
| Categorisation of outcome (if more than one exposure measured, list for each) | Continuous | | |  | Categorical | |  | Quintiles |  | Other | |  | | NA |  | |
| Comments |  | | | | | | | | | | | | | | | |

| Main findings (Free text) |  |
| --- | --- |
| e.g. Higher methylation levels in x CpG site in subjects with low compared with high birthweight (mean ± SD; pvalue); Higher methylation in x CpG associated with lower SEP (effect estimate (95%CI); r=, pvlaue) . Extract every result and document confounder adjustments | |

| Any other comments RE study quality e.g. statistical issues, recall bias, confounder adjustment, generalisability |
| --- |
|  |

| Additional notes |
| --- |
|  |
